# Supplementary figures and images for: Quantitative Raman Analysis of Carotenoid Protein Complexes in Aqueous Solution
Source: Molecules. 2022 Jul 24;27(15):4724. doi: 10.3390/molecules27154724 (PMC9329867; doi:10.3390/molecules27154724)

Figure S1: 1mg/ml Aqueous Carotenoid:BSA Solutions (a) Beta Carotene (b) Lutein (c) Zeaxanthin

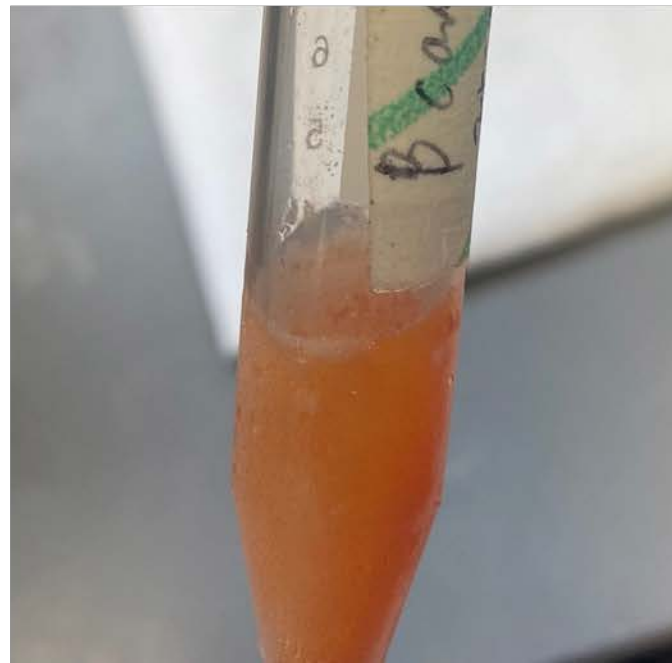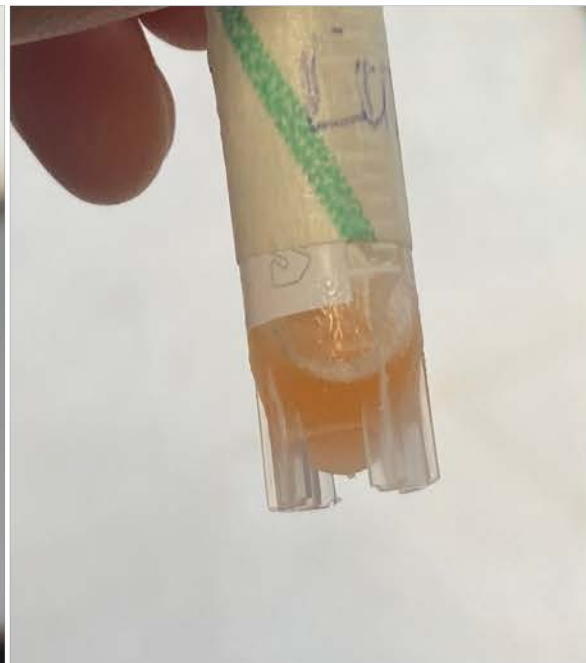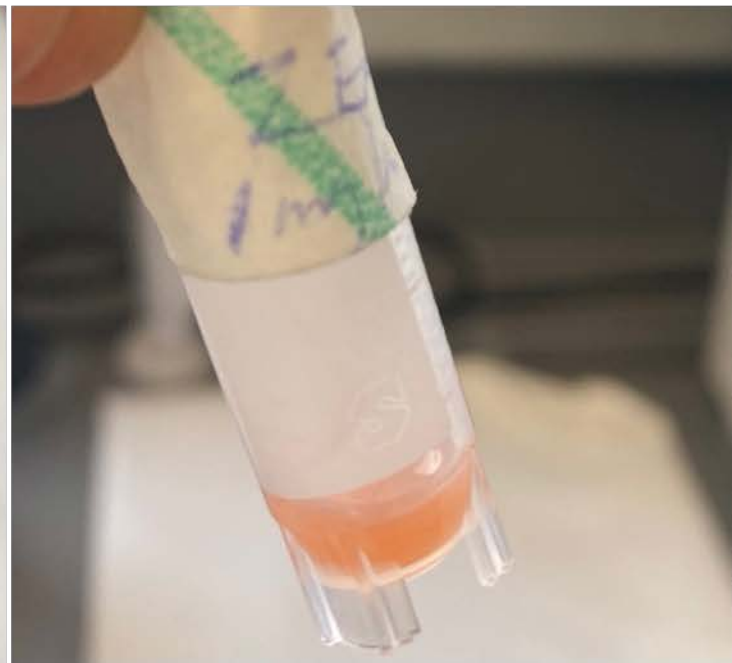

Supplement: Supplementary file 1 [file molecules-27-04724-s001.zip › Figure S1.pdf]

Figure S2: Concentration Dependence of the 'as measured' absorption spectrum of Beta Carotene

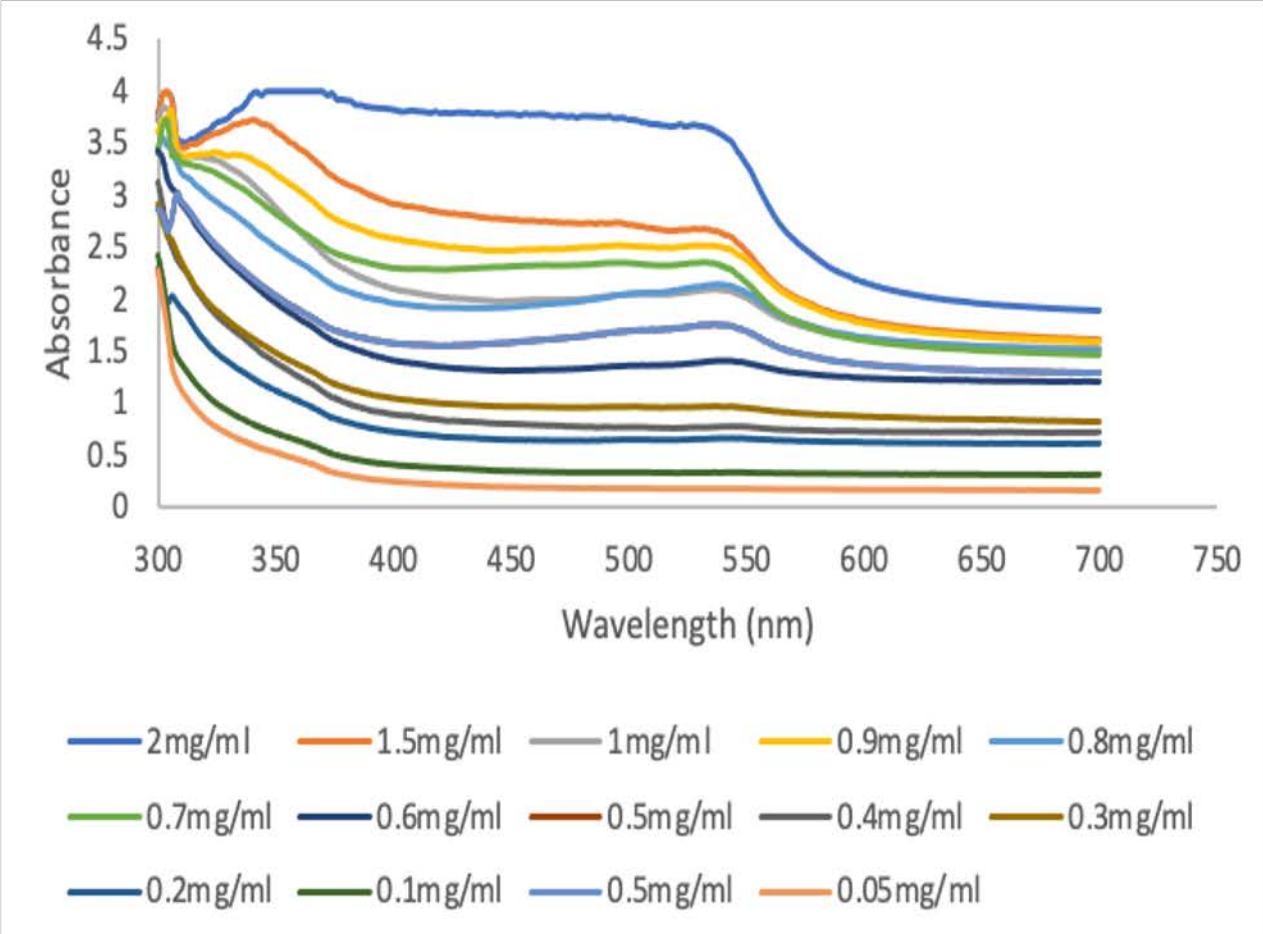

Supplement: Supplementary file 1 [file molecules-27-04724-s001.zip › Figure S2.pdf]
